# High Efficiency Diffusion Molecular Retention Tumor Targeting

**DOI:** 10.1371/journal.pone.0058290

**Published:** 2013-03-11

**Authors:** Yanyan Guo, Hushan Yuan, Hoonsung Cho, Darshini Kuruppu, Kimmo Jokivarsi, Aayush Agarwal, Khalid Shah, Lee Josephson

**Affiliations:** 1 Department of Radiology, Center for Translational Nuclear Medicine and Molecular Imaging, Massachusetts General Hospital, Charlestown, Massachusetts, United States of America; 2 Martinos Center for Biomedical Imaging, Massachusetts General Hospital, Charlestown, Massachusetts, United States of America; 3 Department of Neurobiology, A. I. Virtanen Institute, University of Eastern Finland, Kuopio, Finland; 4 Molecular Neurotherapy and Imaging Laboratory, Department of Radiology, Massachusetts General Hospital, Charlestown, Massachusetts, United States of America; Genentech, United States of America

## Abstract

Here we introduce diffusion molecular retention (DMR) tumor targeting, a technique that employs PEG-fluorochrome shielded probes that, after a peritumoral (PT) injection, undergo slow vascular uptake and extensive interstitial diffusion, with tumor retention only through integrin molecular recognition. To demonstrate DMR, RGD (integrin binding) and RAD (control) probes were synthesized bearing DOTA (for ^111^ In^3+^), a NIR fluorochrome, and 5 kDa PEG that endows probes with a protein-like volume of 25 kDa and decreases non-specific interactions. With a GFP-BT-20 breast carcinoma model, tumor targeting by the DMR or IV methods was assessed by surface fluorescence, biodistribution of [^111^In] RGD and [^111^In] RAD probes, and whole animal SPECT. After a PT injection, both probes rapidly diffused through the normal and tumor interstitium, with retention of the RGD probe due to integrin interactions. With PT injection and the [^111^In] RGD probe, SPECT indicated a highly tumor specific uptake at 24 h post injection, with 352%ID/g tumor obtained by DMR (vs 4.14%ID/g by IV). The high efficiency molecular targeting of DMR employed low probe doses (e.g. 25 ng as RGD peptide), which minimizes toxicity risks and facilitates clinical translation. DMR applications include the delivery of fluorochromes for intraoperative tumor margin delineation, the delivery of radioisotopes (e.g. toxic, short range alpha emitters) for radiotherapy, or the delivery of photosensitizers to tumors accessible to light.

## Introduction

We introduce a technique termed diffusion molecular retention (DMR) tumor targeting which exploits recently developed PEG-fluorochrome shielded probes [1] that, after a peritumoral (PT) injection, undergo extensive diffusion through the interstitium, with tumor retention only through molecular recognition. By exploiting a PT injection and interstitial diffusion, DMR bypasses the many delivery barriers to solid tumors.

Delivery of radiotoxic or chemotoxic “warheads” by antibodies or peptides, and administered by the IV method, is limited by high normal organ uptake and dose-limiting normal organ toxicities. Delivery barriers include tumor hydrostatic pressure [2], perivascular intratumoral concentration [3], [4], targets common to tumor and normal organs, and low tumor blood flow (relative to normal organs). The inability to efficiently target tumor masses is common to antibody and peptide conjugates, though these differ in size and pharmacokinetics. Antibody-based targeting is limited by high hepatic uptake, while peptide targeting is limited by their rapid renal elimination and high retention by the kidney. Efforts to improve IV tumor targeting include multiple drug, pre-targeting strategies [5], [6], multidrug antibody directed prodrug therapies [7], infinite affinity antibodies [8], [9] and increases in antibody valency [10], [11]. Two radiolabeled antibodies have been approved for the treatment of diffuse non-Hodgkin’s lymphoma (Bexxar, Zevalin), but five other radiolabeled monoclonal antibodies, in advanced clinical trials since 2004 [12], have not been approved. Approved antibody drug conjugates (e.g. Myotarg, now withdrawn, and Adcetris) are also indicated for disseminated leukemias or lymphomas, though some designed to target solid tumors are in clinical trials [13]. With radiolabeled peptides, methods to improve the targeting include sequence alteration [14]–[16], multivalency to increase affinity [17]–[20], increasing hydrophilicity to decrease nonspecific organ uptake [21]–[23], and the co-injection of amino acids to limit renal uptake [24].

**Figure 1:**
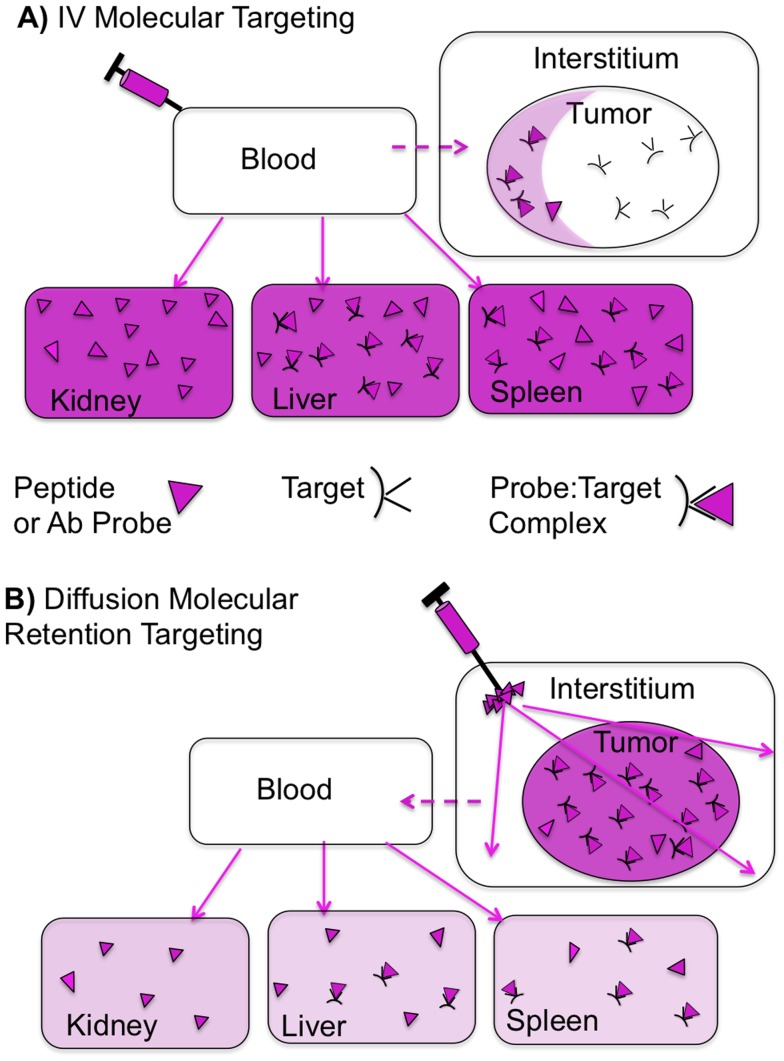
IV Molecular Targeting And Diffusion Molecular Retention (DMR) Molecular Targeting. (A) IV. Retention can be due to target binding, when the probe (triangle) binds to a molecular target (black), or it can be targetless (e.g. kidney Non-specific binding). Non-tumor organs have higher probe concentrations (darker shading) than the tumor. Transport from the vascular compartment (blood) to tumor interstitium (dotted line) is slow while probe transport to normal organs (solid lines) is fast. When the probe reaches the tumor, distribution is uneven (perivascular accumulation). (B) DMR employs a peritumoral (PT) administration, followed by extensive diffusion through normal and tumor interstitium, and retention only if the probe encounters a molecular target. Because the tumor “sees” the agent first, uptake by normal organs is greatly reduced. To obtain extensive interstitial diffusion, transport from the tumor interstitium to the vascular compartment (dotted arrow) must be slow. Slow interstitial to vascular transport results from probe size and hydrophilicity.

**Figure 1A** depicts a typical biodistribution after an IV injection of a peptide or antibody probe. Higher organ uptake is shown as more darkly shaded organs, with normal organ uptake being either largely target-mediated (e.g. RGD probes binding integrins expressed in the liver and spleen) or a non-target mediated (e.g. as in the kidney). Delivery barriers between the vascular compartment and solid tumor produce high probe concentrations in normal organs, a low tumor concentration, and a perivascular intratumor distribution.

Diffusion Molecular Retention (DMR, **Figure 1B**) bypasses the delivery barriers encountered with IV administration, by employing the peritumoral (PT) administration of a probe designed for interstitial diffusion, followed by probe retention thorough binding a molecular target expressed cells of a tumor. DMR consists of (i) the PT injection of a probe, (ii) observing probe diffusion through the normal and tumor interstitium by near infrared (NIR) fluorescence and, (iii) obtaining retention only if the probe encounters a molecular target expressed on cells within the tumor. PT injection exploits the high interstitial diffusion of DMR probes (Figure 1B) and enables the technique in situations where the location of a tumor mass is not precisely known.

To demonstrate the principle of DMR, we employed a recently developed PEG-fluorochrome shielded RGD probe [1]. PEG-fluorochrome shielding is a PEGylation chemistry that blocks the non-specific probe interactions with components of the interstitium, while permitting molecular RGD/integrin interactions, see below. Key features of probe design enabling DMR are discussed further below.

The low dose and ease of synthesis of DMR probes can facilitate the translation of the DMR technique to clinical settings. The efficiency of tumor targeting, and reduction of probe uptake by normal organs, allows the use of low probe doses, minimizing toxicity risks. In addition, DMR probes are synthesized from commonly available raw materials (e.g. Fmoc amino acids, PEG’s, fluorochromes) by high efficiency reaction.

DMR will not be used with curative early stage resections or with metastatic disease where effective IV or oral chemotherapies would be preferred. Rather, DMR is a post-tumor identification, molecular targeting method to be used when a tumor is invasive, and resection is either impossible or possible only with high functional loss. Tumors and organs of origin in this class include those of the pancreas (often inoperable), muscle (sarcoma patients facing possible limb amputation), breast (patients facing possible mastectomy), prostate (patients facing possible prostatectomy), and head and neck cancers (patients facing possible jaw bone removal or larynx removal). In such settings DMR can be used as a targeting technique for either diagnostic or therapeutic agents. Applications of DMR include the delivery of fluorochromes for intraoperative tumor margin delineation, the delivery of radioisotopes (e.g. toxic, short range alpha emitters) for radiotherapy, or the delivery of photosensitizers with tumors accessible to light.

## Results

**Figure 2:**
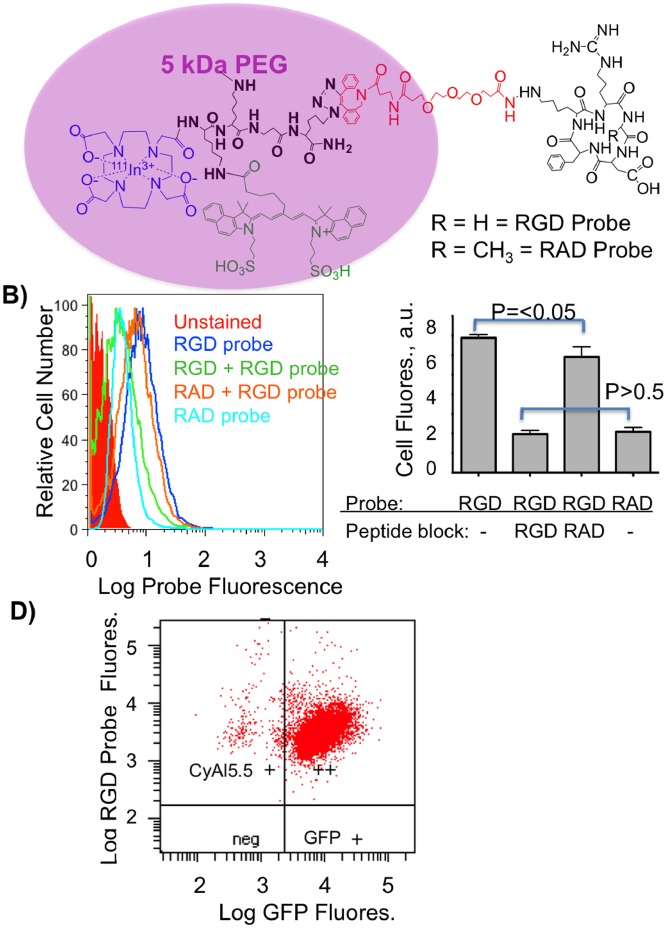
Design of RGD and RAD Probes and their binding to integrins on GFP-BT-20 cells. A) Structures of the RGD and RAD probes. A 5 kDa PEG provides most of the probe volume which is a protein-like 25 kDa by size exclusion chromatography, but does not surround the RGD peptide which binds integrins, see (2B) below. B) Binding of RGD and RAD probes to GFP-BT-20 cells, and displacement by RGD and RAD peptides, by single channel FACS. Also shown is the intrinsic fluorescence of “unstained” calls. C) Displacement of RGD and RAD probes by RGD and RAD peptides. D) Dual wavelength FACS scatter plot for lentivirus transduced, GFP expressing BT-20 cells. Cells bind the RGD probe and express GFP.

To illustrate DMR, we employed an integrin binding RGD probe and a control RAD probe depicted in **Figure 2A** and described earlier [1]. A brief description of the synthesis provided in Materials and Methods below. The 5 kDa PEG forms a diffuse cloud around the fluorochrome, providing PEG-fluorochrome shielding, while an essential short PEG linker (red) enables RGD/integrin binding. The 5 kDa PEG endows the probes with a volume of 25 kDa by size exclusion chromatography using globular protein standards, a volume comparable to small proteins (e.g. Fv = 12 kDa, scFV = 25 kDa) [25]. A volume of this size was expected with a 5 kDa PEG, since PEG’s occupy volumes far larger than indicated by their molecular weights [26]. To verify the role of the 5 kDa PEG in determining probe volume, RGD and RAD probes were synthesized without the 5 kDa PEG and had volumes of less than 1 kDa (see Figure 2e or Table 1 of [1]. The physical properties of RGD and RAD probes employed here are summarized in **Table 1** **.**

**Table 1:** Physical Propoerties of Integrin Targeted and Control Probes.

	TargetPeptide	MW(Da)	MS Obs.(Da)	Equiv.Vol. (kDa)
RGD probe	RGD	7987.4	7980 (peak)	25
RAD probe	RAD	8001.4	8000 (peak)	25

The binding of RGD and RAD probes to integrins, and their displacement with the cyclic RGD or RAD peptides employed in probe synthesis (see **Figure 2A**, black cyclic peptides), was observed with single channel FACS as shown in **Figure 2B**. Probe binding was assessed with BT-20 cells, which bind RGD peptides and antibodies to the α_v_β_3_ integrin [27], [28]. Also shown is the intrinsic fluorescence from “unstained” cells, which was subtracted to obtain the net fluorescence due to probe binding. Median cell fluorescence from **Figure 2B** are then shown in **Figure 2C** **.** The fluorescence from RGD probe binding was blocked by the non-fluorescent RGD peptide, falling to the same fluorescence as was obtained with the control RAD probe (p>0.5). A small but statistically significant (p<0.05) displacement of the RGD probe was seen with RAD peptide. Thus, the RGD and RAD probes differ by a single methyl group (15 daltons), out of total molecular weights of about 8000 Daltons **(** **Table 1** **)** but differ profoundly in their ability to bind integrins. Hence, the specificity of integrin binding *in vivo* can be taken as the difference between RGD probe binding and RAD probe uptake, as we did below, and which has also been done by others [29]–[31]. To enhance the ability to visualize tumors obtained with BT-20 xenografts, GFP-BT-20 cells were obtained through lentivirus transduction as shown in **Figure 2D**.

**Figure 3:**
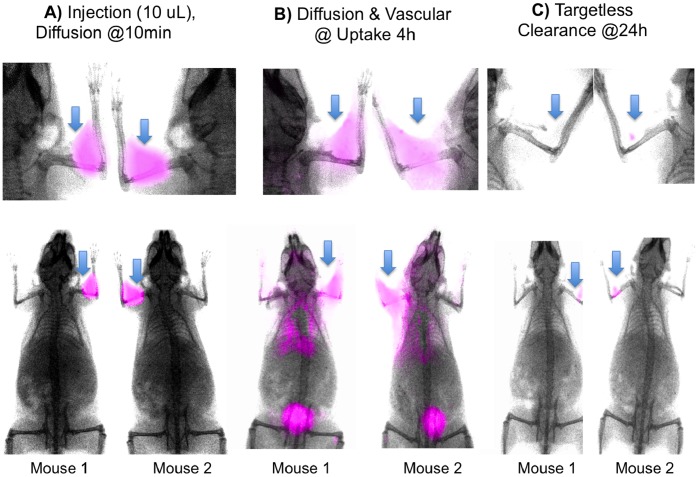
Visualizing of probe interstitial diffusion by surface fluorescence. Diffusion of the control RAD probe after a single IM injection (arrows) into the front extremities (50 pmole/10 µl/injection) are shown. Times post injection were 10 min (A), 4 h (B) and 24 h (C). Injection sites (arrows) show a lack of fluorescence at the injection site at the clearance phase.

Important for molecular targeting with the DMR technique is a combination of extensive interstitial diffusion and lack of non-specific probe retention by components of the interstitium. **Figure 3** shows the time course of diffusion and elimination of the non-integrin binding RAD probe after an IM administration in the front extremities of two nude mice. By surface fluorescence, the probe rapidly diffused through the extremity, shoulder, and thorax on the side of the injection **(** **Figure 3A** **),** with renal elimination evident from bladder fluorescence at 4 h post injection **(** **Figure 3B** **).** Remarkably, by 24 h post injection, probe fluorescence at the injection site was at trace amounts **(** **Figure 3C** **).**

**Figure 4:**
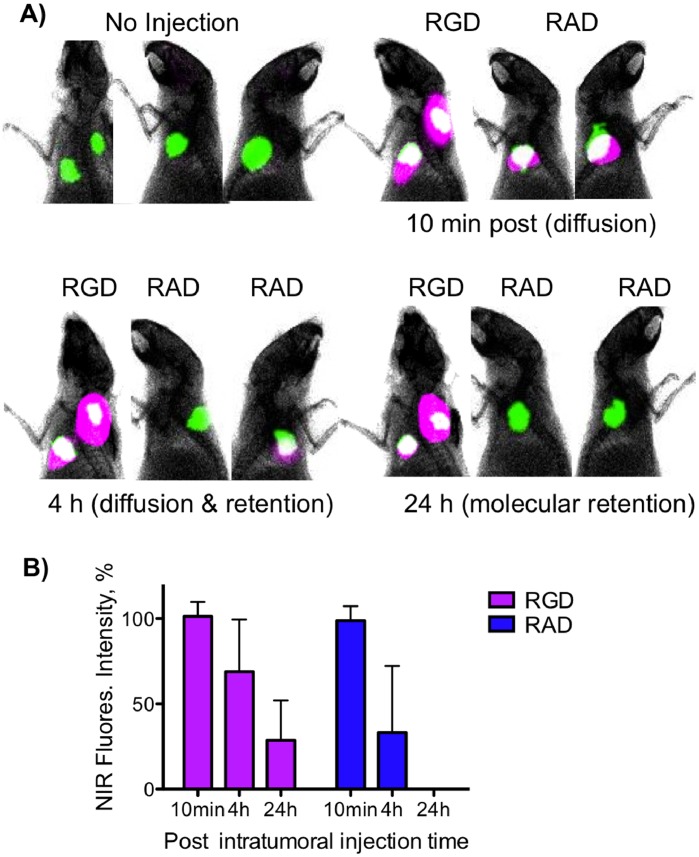
Tumor targeting by DMR by using the GFP expressing BT-20 breast carcinoma xenograft visualized by surface fluorescence. A) Two animals bearing two tumors were PT injected with the RGD probe or RAD probe as indicated and surface fluorescence images were obtained. With the RAD injected animal, tumors were more sagittal so two views of the same animal are provided. Green = GFP. Purple = probe. White = green+purple overlay. The RGD probe diffused around the tumor and is retained while the RAD probe was eliminated. B) Quantitation of tumor surface fluorescence after injections of the RGD or RAD probes as above. Surface fluorescence was quantified through the use of standard solutions. Only the RGD probe was retained by the tumor. n = 4, values are mean ± SD.

The molecular recognition based retention required by the DMR technique is shown in **Figure 4** **.** Shown are two animals (two tumors per animal), with one animal, whose GFP-BT-20 tumors were more sagittal, shown with two views. Prior to injection, tumor borders were assessed by GFP fluorescence, and ten microliters of the RGD probe (or control RAD probe, both at 50 pmoles/injection) was injected between 1 and 2 millimeters from the GFP defined tumor border, a technique referred to as a “peritumoral” (PT) injection. Surface fluorescence images of probe and GFP fluorescence were obtained (10 min, 3 h, 24 h post injection), with the overlaying of probe purple NIR fluorescence on GFP green fluorescence yielding a white color. Tumor surface fluorescence from probes was taken as a region of interest defined by GFP fluorescence, and quantified by the use of solution standards with the means ±1 SD shown in **Figure 4B**. Probes diffused through and around tumors by 10 minutes post injection (**Figure 4A**), with a partial clearance of the RAD probe at 3 h and complete clearance by 24 h. In contrast, the RGD probe was still retained by tumor at 24 h, though it differed from the RAD probe by a single methyl group out of a total molecular weight of about 8000 Daltons **(** **Table 1** **).** At 24 h, tumor fluorescence with the RGD probe was some 28.6±23% of that at 10 min while being undetectable with the RAD probe. With the RGD probe retention occurred, not only within the GFP tumor defined area, but also in a stromal zone surrounding the tumor. When employed with a PT injection, our probe design enables rapid interstitial diffusion around and through the tumor, with retention solely due to integrin interactions, the situation depicted in **Figure 1B** **.**

**Figure 5:**
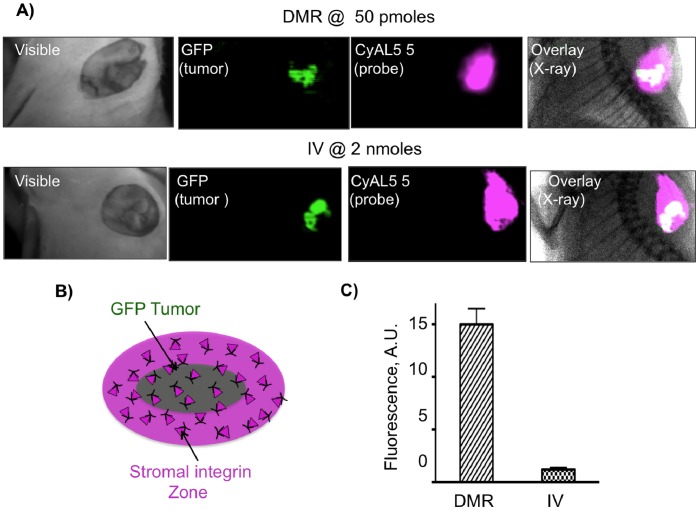
Efficiency of tumor targeting by DMR or IV methods by surface fluorescence. A) Skin covering GFP-BT-20 tumors was removed. Shown are white light images, GFP fluorescence images, probe NIR fluorescence, and the overlay of GFP and probe fluorescence, plus an X-ray image. As with Figure 4, green GFP plus purple NIR fluorescence yields a white overlaid image. B) By with PT DMR or IV, probe fluorescence included a stromal zone of integrin binding surrounding the tumor as was seen in (a). C) A comparison of tumor surface fluorescence intensities by PT DMR versus the IV methods is shown. Doses were 50 pmoles (per site) and 2000 pmoles (IV) for figures 5 and 6.

**Figure 6:**
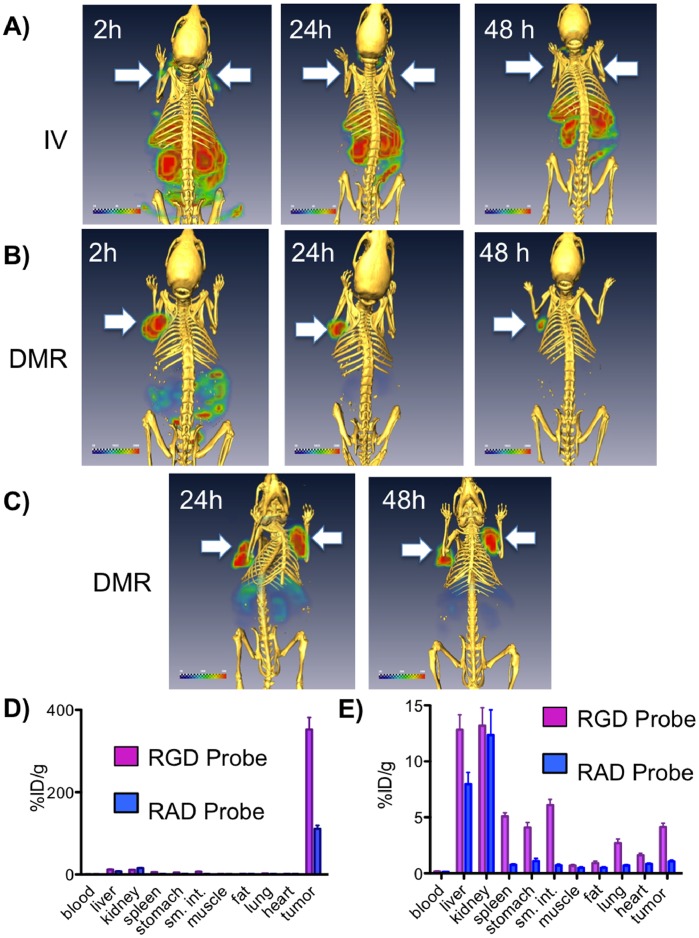
SPECT/CT images after PT and IV injections with the ^111^In RGD probe using the BT-20 tumor model. SPECT images after injections (A–C) of the ^111^In-RGD probe by the IV or PT DMR methods are shown with one or two tumors/animal (arrows). Radioactivity is shown with a green to red color scale, while CT bone density is yellow. A) Tail vein IV injection. B) Single PT injection (DMR). C) Dual PT injections (DMR). Post dissection tissue radioactivity concentrations obtained with the ^111^In-RGD and ^111^In-RAD probes by IV injection (D) and PT injection (E) are shown. Radioactivity was 0.3 mCi per injection for IV and PT injections in this figure.

To compare the efficiency of tumor targeting with the DMR and IV methods in setting similar to that encountered surgical tumor resection, surface fluorescence images of tumor GFP and RGD probe fluorescence were obtained with skin removed, as shown in **Figure 5**. With both DMR and IV administration, probe fluorescence extended beyond tumor GFP margins to a stromal area beyond the tumor **(** **Figures 5A, 5B** **),** consistent with **Figure 4A** **.** However, tumor fluorescence **(** **Figure 6C** **)** was far higher with DMR (15.0±1.5 A.U.) than IV injection (1.2±0.2), even though dose by the PT DMR method (50 pmoles/mouse) was forty times lower (2000 pmoles/mouse by IV).

To further compare probe biodistributions by the DMR and IV methods, SPECT-CT images with the ^111^In labeled RGD probe as shown in **Figures 6A–6C**. With IV administration at 2 h post injection (**Figure 6A**), radioactivity was predominantly in the liver, kidney and small intestine, with limited tumor radioactivity (arrows). Radioactivity in the lower abdomen was from the stomach and small intestine based on the post IV dissection studies shown on **Figure 6E**. With a single PT administration (**Figure 6B**), radioactivity was largely in the tumor at 2 h post injection and exclusively in the tumor by 24 h. DMR images at 24h post injection with dual PT injections are shown in **Figure 6C** **.**

Tissue concentrations were then obtained with the IV and DMR methods using an ^111^In- labeled RGD probe and an ^111^In RAD probe as shown in **Figures 6D and 6E**. With the RGD probe tumor radioactivity was 352±41%ID/g by DMR versus 4.1±1.1%ID/g by IV administration. The higher binding of the RGD probe versus the RAD probe indicates integrin specific uptake of the RGD probe, which occurred in normal organs such as the stomach, small intestine and spleen, as well as in the tumor (Figure 6E). By using a PT injection, DMR enhances RGD/integrin molecular tumor targeting and reduces RGD/integrin targeting that occurs with integrin expressing, normal tissues and IV injection.

## Discussion

We employed three techniques (surface fluorescence, SPECT, post dissection ^111^In-biodistribution) to demonstrate that DMR is an efficient method of tumor targeting, compared to the relatively inefficient targeting obtained with IV administration. To accomplish this, we employed the well-studied interaction of RGD peptides with integrins, in part because of ability to synthesize a control RAD probe and demonstrate the interstitial diffusion and clearance from the injection site in the absence of molecular interactions (e.g. **Figures 3** and **4**). The higher efficiency of tumor targeting obtained with DMR, relative to the standard IV method, was evident by tumor surface fluorescence (**Figure 5**), with SPECT imaging (**Figure 6**) and biodistribution studies (**Figure 6**). Surface fluorescence was 15.0 A.U. by DMR but only 1.2 A.U. by IV (**Figure 5C**), while tissue radioactivity was 352% ID/g by DMR but only 4.1%ID/g by IV (**Figures 6D, 6E**).

For efficient molecular retention targeting following the PT injection used by DMR (**Figures 4** and **5**), three conditions of probe design and performance must be met. *First,* the probe must bear a fluorochrome so that diffusion through the interstitium can be observed. NIR fluorochromes are desirable because of the tissue penetrating properties of NIR light. However, NIR fluorochromes typically involve extended unconjugated double bond systems connecting two unsaturated rings, and these features exacerbate non-specific interactions. Therefore *second,* probe design must employ PEG-fluorochrome shielding, to block non-specific binding (particularly that mediated by the fluorochrome) to the interstitium after IM (**Figure 3**) or PT injections (**Figure 4**). A comparison of probes (**Figure 2A**) with and without the 5 kDa PEG functional group demonstrates the key role of the 5 kDa PEG plays in clearance from the injection site after an IM injection (Figure 3 of [1]). *Third,* probe transport from the interstitial space to the vascular compartment (blood) must be slow (**Figure 1B** dotted arrow), providing the time needed for extensive interstitial diffusion. The 5 kDa PEG increased probe volume to that of a small protein (25 kDa), and conferred a highly hydrophilic character upon the probe. Both size and hydrophilicity slow the rate of interstitial to vascular compartment transport [32], and enhance the time available for interstitial diffusion.

A variety of minimally invasive or local injection techniques might permit the PT injection required by DMR for tumors in a variety of anatomical settings. Local injection techniques are used for sentinel lymph node determination, treating benign prostatic hyperplasia [33], [34], treating urinary incontinence [35], [36], and for stem cell delivery [37]. Invasive basal cell skin carcinomas would be also amenable to a PT injection.

Though our goal was to demonstrate DMR as a high efficiency molecular tumor targeting technique, two potential clinical applications of DMR are suggested; they are the delivery of fluorochromes to invasive tumors for intraoperative margin delineation and the delivery of toxic radioisotopes to tumors for radiotherapy. For margin delineation, the efficient delivery of probes can greatly reduce both reagent costs and systemic toxicity risks. The efficiency of DMR targeting is apparent from the dose we employed, which was 50 pmoles/injection site (3.0 ng as RGD peptide), and which would correspond to a dose of only 8.4 µg of peptide for a 70 kg human. Clinically, multiple PT injection sites and larger injection volumes (0.1 to 0.5 mL versus the 10 µL used here) may enable probes to diffuse through larger and more varied human tumors. With respect to margin delineation and breast cancer, the RGD sequence might prove suitable, since with tumor microarray specimens ductal and lobular carcinomas bind RGD peptides [38]. In addition, local delivery techniques are used to administer compounds for sentinel lymph node determination for breast cancer.

A second attractive class of DMR applications is the delivery of therapeutic radioisotopes to invasive, pre-metastatic tumors. Here, DMR offers the delivery of high radiation doses to tumors and with greatly reduced radiation burdens to normal organs. The DOTA functional group can chelate a range of trivalent metals SPECT or PET (^111^In, ^68^Ga) [39] or for radiotherapy (e.g. ^213^Bi [40], [41], ^177^Lu [42], [43], ^90^Y [42], [44], [45] or ^225^Ac [46], [47]. DMR maybe particularly well suited to the delivery of alpha particle emitters, with their high toxicity and short range of action [12], [48].

DMR method has been developed, not only to provide a method for high efficiency tumor targeting, but also to provide a technique that is amenable to clinical translation. While achieving the volume of small proteins, DMR probes are not biologicals. In addition, DMR tumor targeting permits the use of low probe doses, such as the 25 ng (as RGD peptide) employed here, which may reduce toxicity risks. The history of difficulties with the targeted, molecular delivery of “payloads” to solid tumors by IV administration, (see **Figure 1A** and the related discussion), coupled with the enormous costs of IV administered antibodies (or antibody-conjugate drugs), may make the DMR technique practical in the ever more cost-conscious world of cancer treatment likely to prevail over the coming decades.

## Materials and Methods

**Figure 7:**
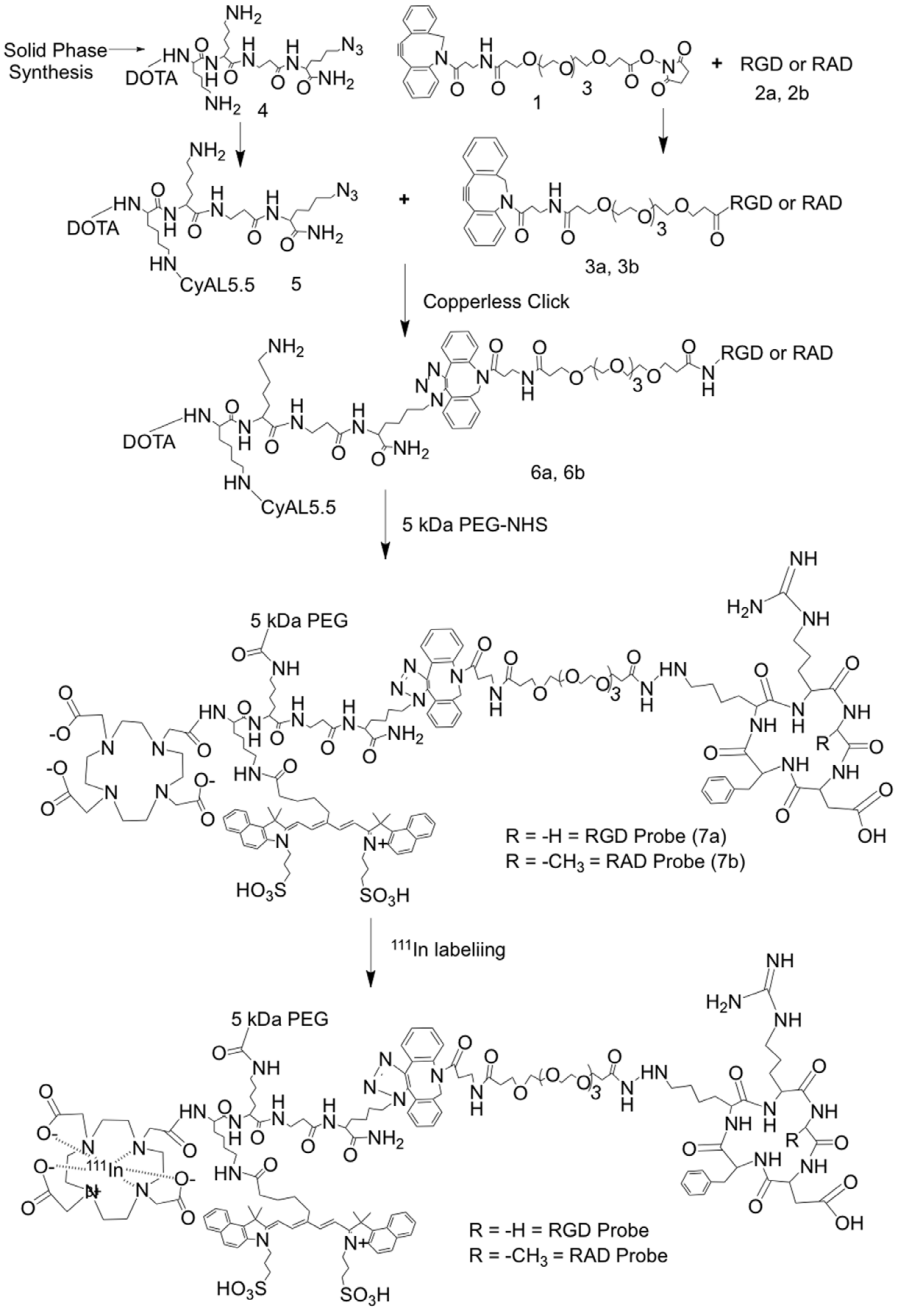
Synthesis of RGD and RAD probes. The general strategy used to synthesize the RGD probe (7a) and RAD probe (7b) is shown.

The synthesis and characterization of the RGD and RAD probes employed here, and whose structures are given in **Figure 2A**, is given in detail in [1]. The RGD probe here is compound 7a while the RAD probe is compound 7b from [1]. A brief description of the synthesis of these probes is provided, with an outline of steps employed shown in **Figure 7**. The CyAL5.5 fluorochrome, a variant of Cy5.5 with similar absorption and emission maxima, was synthesized as described [49]. Probe synthesis employs a multifunctional single attachment point (MSAP) reagent strategy, where a variety of functional groups are attached to peptide scaffold and then reacted with targeting RGD or RAD peptides [50]–[52]. As shown in **Figure 7**, two functional groups (DOTA, CyAL5.5) were attached to a Lys-Lys-βAla-Lys(N_3_) peptide scaffold on a solid phase, to yield an MSAP reagent which in peptide notation is (DOTA)Lys(CyAL5.5)-Lys-βAla-Lys(N_3_). This compound, 5 from [1], was purified by reverse phase HPLC. Separately, the epsilon lysine amines of RGD and control RAD targeting cyclic pentapeptides (cyclic RGDfK, cyclic RADfK from Peptides International) were reacted with a linker of DBCO-PEG4-NHS (Click Chemistry Tools), yielding compounds 3a, 3b. DBCO is dibenzylcyclooctyne. 3a or 3b were then reacted with the azide of the MSAP reagent (5) using a copperless click reaction between the DBCO group and the azide on the MSAP reagent, to yield compounds 6a and 6b. Finally reaction with NHS-5 kDa PEG (from Creative PEGworks) yielded the RGD (7a) and RAD (7b) probes. Probes were single peaks of 25 kDa by FPLC (ÄKTA Purifier 10 and Superdex™ 75 10/300GL column (GE Healthcare)), with molecular weights of 7980 and 8000 Da by Mass Spectra (MS-ESI Micromass (Waters) and MALDI-TOF analyses).

^111^In radiolabeling RGD and RAD probes was as described in [1]. Radiochemical purity was >95%.

BT-20, a human breast carcinoma cell line, highly expressing integrin, was from the American Tissue Culture Collection and maintained according to their instructions. Probe binding assay and protocol for transfecting BT-20 with GFP can be seen in supporting information.

Female nude mice (25–30 g; 6–8 weeks old; nu/nu) were anesthetized with 2% isoflurane/O_2_. GFP-BT-20 cells were detached, pelleted and 200 µl of cell suspension containing 10^6^ cells in Matrigel (BD) was injected subcutaneously into right and left shoulders. Tumors were allowed to grow 7 to 10 days before experiments. All animal experiments were approved by the Institutional Review Committee of the Massachusetts General Hospital, protocol number 2009N000043. Tumor implantation was performed with ketamine/xylazine and all efforts were made to minimize suffering.

A Kodak FX multispectral imaging system was used (Carestream Molecular Imaging, Rochester, NY) for surface fluorescence imaging. Detailed protocol can be found in supporting information.

The *SPECT/CT* imaging was performed by Triumph II multimodality imaging system (Gamma Medica Ideas, LLC) comprising XSPECT with four CZT (Cadmium Zink Telluride) detectors and X-O CT with CMOS detector. SPECT data of the ^111^In-labeled compound was acquired for 60 min using 5-pinhole collimators and processed with 3D-OSEM algorithm using 4 subsets and 5 iterations. 3-dimensional CT data was processed with modified Feldkamp software. The processed 3D-images were fused and displayed with VIVID software package installed to the Triumph data management. Animals were under isoflurane anesthesia (1.5%) with O_2_ flow (1.5 l/min) and kept warm during the imaging with a heated animal bed.

*Biodistribution of ^111^In labeled RGD or RAD probes:* 150 µl of ^111^In-labeled RGD probe/7a or RAD probe/7b (300 µCi, ∼50 pmole) were injected to tumor-bearing animals by tail vein (IV). 24 h later, animals were sacrificed, and tumors, blood, liver, spleen, stomach, kidneys, small intestine, lung, heart, tail, fat, and muscle, were collected. Radioactivity was measured with Perkin Elmer, Wizard2 2480 gamma counter.

## Supporting Information

File S1 (DOCX)
