# Supplementary material for: High Efficiency Diffusion Molecular Retention Tumor Targeting
Source: PLoS One. 2013 Mar 11;8(3):e58290. doi: 10.1371/journal.pone.0058290 (PMC3594319; doi:10.1371/journal.pone.0058290)
Supplement: File S1 — (DOCX) [file pone.0058290.s001.docx]

Supporting information for

"High Efficiency Diffusion Molecular Retention Tumor Targeting"

Yanyan Guo, Hushan Yuan, Hoonsung Cho, Darshini Kuruppu, Kimmo Jokivarsi, Aayush Agarwal, Khalid Shah, Lee Josephson

**Materials and Methods**

The synthesis and characterization of the RGD and RAD probes employed here, and whose structures are given in Figure 2A, is given in detail in [[1](#_ENREF_1)]. The RGD probe here is compound 7a while the RAD probe is compound 7b from [[1](#_ENREF_1)]. A brief description of the synthesis of these probes is provided, with an outline of steps employed shown in Figure 7. The CyAL5.5 fluorochrome, a variant of Cy5.5 with similar absorption and emission maxima, was synthesized as described [[49](#_ENREF_49)]. Probe synthesis employs a multifunctional single attachment point (MSAP) reagent strategy, where a variety of functional groups are attached to peptide scaffold and then reacted with targeting RGD or RAD peptides [[50-52](#_ENREF_50)]. As shown in Figure 7, two functional groups (DOTA, CyAL5.5) were attached to a Lys-Lys-βAla-Lys(N_3_) peptide scaffold on a solid phase, to yield an MSAP reagent which in peptide notation is (DOTA)Lys(CyAL5.5)-Lys-βAla-Lys(N_3_). This compound, 5 from[[1](#_ENREF_1)], was purified by reverse phase HPLC.

Separately, the epsilon lysine amines of RGD and control RAD targeting cyclic pentapeptides (cyclic RGDfK, cyclic RADfK from Peptides International) were reacted with a linker of DBCO-PEG4-NHS (Click Chemistry Tools), yielding compounds 3a, 3b. DBCO is dibenzylcyclooctyne. 3a or 3b were then reacted with the azide of the MSAP reagent (5) using a copperless click reaction between the DBCO group and the azide on the MSAP reagent, to yield compounds 6a and 6b. Finally reaction with NHS-5 kDa PEG (from Creative PEGworks) yielded the RGD (7a) and RAD (7b) probes. Probes were single peaks of 25 kDa by FPLC, with molecular weights of 7980 and 8000 Da by Mass Spectrometry.

^111^In radiolabeling RGD and RAD probes was as described in [[1](#_ENREF_1)]. Radiochemical purity was >95%.

*Probe characterization:* Molecular weights were obtained by MS-ESI Micromass (Waters) and MALDI-TOF analyses at the Tufts University Core Facility. RP-HPLC (Varian ProStar detector and delivery modules) employed an eluant A (0.1% TFA /water) and eluant B (0.1% TFA in 9.9% water in acetonitrile). Probe size (volume) was determined by FPLC using an ÄKTA Purifier 10 and SuperdexTM 75 10/300GL column (GE Healthcare) with a running buffer of 0.05 M sodium phosphate, 0.15 M NaCl (0.1% Tween, pH 7.2) and flow rate of 0.5 ml/min. Standards (GE Healthcare) were aprotinin, ribonuclease A, ovalbumin, and conalbumin) and Blue Dextran 2000. To obtain probe volumes, Mr (apparent molecular weight based on size exclusion retention) was ploted versus Kav. Kav = (Ve-Vo)/(Vt-Vo), Vt = total volume, Ve = elution volume, Vo = void volume.

*Probe binding to integrin expressing cells*: BT-20, a human breast carcinoma cell line, was from the American Tissue Culture Collection and maintained according to their instructions. Cells were seeded on 12-well plates at 5 X 10^5^ cells/well in culture medium (EMEM with 10% FBS) the day before the assay. The day of assay, medium was removed, wells rinsed twice with DPBS (+Ca, +Mg), and 100 μl of 1% FBS / DPBS (+Ca, +Mg) added. Probe binding was blocked with “RGD” peptide (RGDfK) or an “RAD” peptide (cRADfK). Peptides (350 μM) in 1% FBS / DPBS (+Ca, +Mg) (100 μL/well) was added to cells and incubated for 1h at 37 ^o^C. Then 100μL of RGD probe or control RAD probe (2μM) was added, with incubation for another 30 min. (Probe concentrations were determined spectrophotometrically (694 nm, extinction coefficient of 130,000 cm^-1^ M^-1^ for CyAL5.5 [[1](#_ENREF_1)]. Cells were detached by Trypsin/EDTA and assayed for fluorescence by FACS (BD 3 laser LSR2). The median cell fluorescence after probe exposure was subtracted from the median fluorescence of unstained to provide values of cell fluorescence which were expressed as means ± 1 standard error, n= 3.

*Surface fluorescence imaging*: A Kodak FX multispectral imaging system was used (Carestream Molecular Imaging, Rochester, NY). Excitation at multiple wavelengths (600, 610, 620, 630, and 650 nm) with the emission at 700 nm was setup for CyAL5.5 spectrum; Excitation at multiple wavelengths (440, 450, 460, 470, and 480nm) with the emission at 535nm was setup for GFP spectrum, with manufacturer’s software to separate (unmix) the CyAL5.5 spectrum or GFP spectrum from skin autofluorescence and chlorophyll fluorescence from food. X-ray images were taken after fluorescence images. Animals were immobilized with ketamine/xylazine during imaging. All animal experiments were approved by the Institutional Review Committee of the Massachusetts General Hospital.

*Surface fluorescence for diffusion/retention of the RAD probe with IM injection and normal mice:* Ten microliters of the RAD probe (50 pmoles) were injected intramuscularly at front leg of each mouse and surface fluorescence images obtained.

*Lentivirus transduction for GFP expression in BT-20 cells*. BT-20 cells and DMEM media were from ATCC, while DPBS and FBS were from GIBCO. BT-20 cells were plated on 24-well plate at 750,000 cells/well in culture medium (EMEM with 10% FBS) and transduced with lentivirus (LV)-GFP-Fluc [[53](#_ENREF_53)] (1 X 10^8^ particles/mL) in a fresh culture medium with 2 mg/L of protamine sulphate (American Pharmaceutical Partners, 10 mg/mL). The next day, culture medium (EMEM with 10% FBS) was added and, after an additional 24 h, GFP expression was analyzed by fluorescence microscopy and FACS.

*GFP-BT-20 tumor model*: Female nude mice (25-30 g; 6-8 weeks old; nu/nu) were anesthetized with 2% isoflurane/O_2_. GFP-BT-20 cells were detached, pelleted and 200 µl of cell suspension containing 10^6^ cells in Matrigel (BD) was injected subcutaneously into right and left shoulders. Tumors were allowed to grow 7 to 10 days before experiments. All experiments were approved the MGH committee on animal care.

*Imaging molecular retention by surface fluorescence after peritumoral probe injections using the GFP-BT-20 tumor model:* The RGD probe (or the RAD probe) (50 pmoles, 10 µL) was injected within 1-2 mm of tumors, with margins determined by GFP fluorescence.

*Surface fluorescence after peritumoral and IV injections:* Peritumoral injection of the RGD probe was as above. IV injection of the probe was by tail vein (2 nmoles).

*SPECT/CT*: The imaging was performed by Triumph II multimodality imaging system (Gamma Medica Ideas, LLC) comprising XSPECT with four CZT (Cadmium Zink Telluride) detectors and X-O CT with CMOS detector. SPECT data of the ^111^In-labeled compound was acquired for 60 min using 5-pinhole collimators and processed with 3D-OSEM algorithm using 4 subsets and 5 iterations. 3-dimensional CT data was processed with modified Feldkamp software. The processed 3D-images were fused and displayed with VIVID software package installed to the Triumph data management. Animals were under isoflurane anesthesia (1.5%) with O_2_ flow (1.5 l/min) and kept warm during the imaging with a heated animal bed.

*Biodistribution of ^111^In labeled RGD or RAD probes:* 150 µl of ^111^In-labeled RGD probe/7a or RAD probe/7b (300 µCi, ~50 pmole) were injected to tumor-bearing animals by tail vein (IV). 24 h later, animals were sacrificed, and tumors, blood, liver, spleen, stomach, kidneys, small intestine, lung, heart, tail, fat, and muscle, were collected. Radioactivity was measured with Perkin Elmer, Wizard^2^ 2480 gamma counter.

**References for supplementary materials.**

1. Guo Y, Yuan H, Rice WL, Kumar AT, Goergen CJ, et al. (2012) The PEG-Fluorochrome Shielding Approach for Targeted Probe Design. J Am Chem Soc.

53. Shah K, Hingtgen S, Kasmieh R, Figueiredo JL, Garcia-Garcia E, et al. (2008) Bimodal viral vectors and in vivo imaging reveal the fate of human neural stem cells in experimental glioma model. J Neurosci 28: 4406-4413.
